# It is time to change the way we think about hearing evaluation

**DOI:** 10.1007/s00405-024-08620-1

**Published:** 2024-04-04

**Authors:** Vasiliki Maria Iliadou, Doris-Eva Bamiou, William Keith, Suzanne C. Purdy, Hung Thai-Van

**Affiliations:** 1 Medical School, Aristotle University of Thessaloniki, Thessaloniki, Greece; 2 Ear Institute, Faculty of Brain Sciences, University College London, London, UK; 3 School of Psychology, Faculty of Science, The University of Auckland, Auckland, New Zealand; 4 Hospices Civils de Lyon, Service d’Audiologie & Explorations Oto-Neurologiques, Lyon, France; 5 Institut de L’Audition, Institut Pasteur, Paris, France; 6 Université, Claude Bernard Lyon 1, Villeurbanne, France

**Keywords:** Hearing, Auditory processing, Speech-in-noise, Hearing evaluation, Auditory processing disorder, Pure tone audiogram

Keeping up with current research in any medical scientific area is time consuming. Journals relevant to audiology are numerous and extend beyond the traditional field. Researchers and clinicians evaluating hearing often limit their update to reading of titles, abstracts or conclusions due to time restrictions. This paper aims to discuss common misconceptions about hearing evaluation and provide recommendations for hearing evaluation based on current research.

A recent paper by Goodwin et al. [1], although acknowledging that the gold standard for hearing evaluation is the pure tone audiogram, used self-report alone as the measure of hearing loss in adults. Goodwin et al. found an association of self-reported hearing difficulties with greater memory problems, that was mediated by psychosocial factors. This is just one example of an emerging trend of weighting equally self-reported hearing-related difficulties versus audiometrically documented. Although self-report is important as an auxiliary approach, it should not be used as a standalone tool in light of limited evidence for its validity and its susceptibility to a range of factors, such as personality type [2, 3].

The recent WHO Report on Hearing [4] acknowledged that pure tone audiometry, despite being considered as the gold standard for hearing evaluation, does not provide the best insight into hearing capacity in everyday life situations [5]. Self-report using standardized questionnaires can provide insights into everyday life listening challenges, however, self-report as a sole tool for identifying hearing difficulties could overestimate prevalence, without specifying the nature of the difficulties. In the area of auditory processing disorder (APD), self- or caregiver-reported listening difficulties may be considered the ‘gold standard’ for identification of APD rather than diagnostic assessments with established reliability and validity. For example, Moore et al. [6] captured ‘clinical presentation’ with the Children’s Auditory Processing Performance Scale (CHAPPS) and the Children’s Communication Checklist 2 (CCC-2). A recent research survey [7] with 134 US audiologists shows that only 9% see APD as not being a unique disorder while acknowledging frequent comorbidity. The same survey reports on APD diagnostic protocol consistency as well as referrals to other professionals or multidisciplinary assessment based on case history as the most common approach to comorbidity issues.

A more real-life assessment of hearing capacity would be to include a speech-in-competition (noise/babble) test. This is a key component of auditory processing evaluation in related guidelines [8]. The term auditory processing refers to the efficiency and accuracy with which auditory information is encoded and conveyed from the cochlea across the central auditory nervous system. Auditory processing is typically assessed through a battery of psychoacoustic tests that evaluate different elements of auditory processing such as speech perception in competing noise/babble, dichotic listening, temporal resolution, pitch discrimination and sequencing, and other measures of auditory discrimination. Test selection is driven by a detailed history of self- or caregiver-report of hearing and listening difficulties and a thorough developmental, educational, medical and family history. Auditory processing evaluation takes on average 1 to 2 h to complete and this may partly account for limited clinical service availability and related expertise.

Time is of the essence and a key consideration in current audiological and other clinical settings. Assessments are usually conducted by highly trained hearing professionals using calibrated test equipment in specialised testing environments, hence speed is required to reduce costs and to optimise the use of the professional’s time. The use of rapidly-administered screening techniques is becoming more widespread, facilitated by technological progress. Screening can be carried out in a few minutes and thus may be considered as preferable to diagnostic evaluation when the distinction between screening and diagnosis is unclear.

Although screening is useful in discovering issues in large groups with no suspected problems (for example screening all children in a school, regardless of hearing history or concerns), screening could have low accuracy if implemented in individuals with reported symptoms suggestive of hearing difficulties where a detailed diagnostic approach is required [9]. In the area of APD, screening may be particularly challenging since there are different facets of auditory processing, making it difficult to use just one screening test [10]. Because of the difficulty of comprehensive behavioural screening for APD, a checklist approach is preferred to identify difficulties that highlight the need for diagnostic assessment.

Although the key differences between screening and diagnosis of hearing difficulties are well established [11], there are many examples where this distinction is not being made. As hearing impairment may be a "hidden disability" and comprehensive diagnostic hearing evaluation may not always be considered or selected for a range of reasons [12], it is important to establish guidelines for core hearing evaluations. Based on current research and clinical observations we propose the following.

## Recommendations for hearing evaluation

1. Diagnostic assessment should be conducted for people of all ages presenting with any of the following: reported hearing or communication difficulties, inattention, easy distraction or listening challenges in noisy environments, and speech, language, phonological or learning difficulties. This evaluation should include pure tone audiometry at frequencies 250 Hz, 500 Hz, 1000 Hz, 2000 Hz, 3000 Hz, 4000 Hz, 6000 Hz, 8000 Hz per ear as a minimum gold standard [13] for individuals older than 5 years old. Implementation may be challenging if masking is required or if a child does not comply with alternative approaches including free-field testing or a more playful approach moving cubes from one basket to another every time a sound is heard. Extending pure tone audiometric frequencies testing up to 16 kHz may be required depending on patient history (i.e. during chemotherapy).
2. For younger ages and other cases where pure tone audiometry is challenging, diagnostic otoacoustic emissions (OAEs) and auditory brainstem responses are reliable objective hearing measures. Immittance testing that includes acoustic reflexes and OAEs are rapidly administered objective tests that can be administered across the lifespan.
3. In cases with suspected hearing difficulties but a normal pure tone audiogram, assessments of speech perception in quiet and in noise should take place [14]. Abnormal speech in noise results maybe due to APD, Auditory Neuropathy Spectrum Disorder or Hidden Hearing Loss (HHL) [15] making this test an essential part of hearing evaluation. However, identification of HHL in an individual with normal hearing remains a clinical challenge and requires a combination of electrophysiological and electroacoustic tests and a high clinical acumen in the presence of related risk factors [16].
4. In cases with poor speech perception, or with reported listening difficulties despite a normal audiogram, auditory processing evaluation should be implemented [17]. The minimum APD test battery includes assessments of speech perception in noise/babble, dichotic listening and temporal processing.
5. Questionnaires should also be considered. According to a recent systematic review [2] questionnaires that differentiate APD-diagnosed individuals (based on currently established criteria) from age-matched controls and other clinical groups are: Children’s Auditory Processing Performance Scale (CHAPPS), Fisher’s Auditory Problems Checklist (FAPC), Auditory Processing Domains Questionnaire (APDQ), Speech, Spatial, and Qualities of Hearing Scale (SSQ), Amsterdam Inventory for Auditory Disability and Handicap (AIAD) and Hyperacusis Questionnaire (HQ) for adults. This review stressed the limited evidence for questionnaire subscales that could provide more elaborate information on specific difficulties related to hearing.
6. Cognitive and language factors [18–21] may inform test selection and approach, similar to their determining the appropriate conduct of pure tone audiometry. However, these do not preclude diagnostic assessment for APD. Collaboration with speech pathologists, pediatricians, occupational therapists, psychologists, educators, geriatricians and specialised centers is recommended. Reports from these other disciplines prior to diagnostic audiology can streamline testing and facilitate the choice of assessments. For adults with memory difficulties/suspected cognitive impairment referral to a specialist clinic is recommended. Considerations for further evaluation should be decided on a case-by-case basis.

## Data Availability

Not applicable.
